# Study on the regulatory effect of Panax notoginseng saponins combined with bone mesenchymal stem cell transplantation on IRAK1/TRAF6-NF-κB pathway in patients with diabetic cutaneous ulcers

**DOI:** 10.1186/s13018-022-03467-w

**Published:** 2023-01-31

**Authors:** Yuqing Du, Weijian Chen, Youshan Li, Du Liang, Guobin Liu

**Affiliations:** 1 Peripheral Vascular, Shuguang Hospital, Shanghai University of Traditional Chinese Medicine, Shanghai, 201203 China; 2 Guangzhou University of Chinese Medicine, Guangzhou, 510405 Guangdong Province China; 3 Department of Orthopaedics, Guangzhou Orthopedic Hospital, Guangzhou University of Chinese Medicine, Guangzhou, 510045 Guangdong Province China; 4 Peripheral Vascular, Dongzhimen Hospital, Beijing University of Chinese Medicine, Beijing, 100000 China

**Keywords:** Panax notoginseng saponins, Bone marrow mesenchymal stem cells, Diabetic cutaneous ulcer, Bioinformatics, miR-146a-5p, IRAK1/TRAF6-NF-κB signaling pathway

## Abstract

Panax notoginseng saponins (PNSs) have been found as the major active ingredient of Panax notoginseng (Burkill) F.H.Chen (PN) leaves, which has the effect of reducing inflammatory response, facilitating fibroblast proliferation, as well as promoting angiogenesis. This study aimed to investigate the molecular basis of PNS combined with bone mesenchymal stem cells (BMSCs) for treating diabetic cutaneous ulcers (DCU) and its mechanism of action. Methods. A total of 75 SD rats were selected to make diabetic cutaneous ulcers model. According random number table method, the rats were randomly divided into a control group, a DCU group, a BMSCs group, a PNS group and BMSCs + PNS group. Five groups of rats were given without treatment. After being treated for 7 days, the rats were anesthetized with pentobarbital, and granulation tissue was collected from the central point of the wound. They were used for pathological analysis, Western blot (WB) and polymerase chain reaction (PCR) assays. Results. The wound healing area was the largest in the BMSCs + PNS group. HE staining results showed that the PNS + BMSCs group could promote the formation of new epidermis and reduce the infiltration of inflammatory cells. Immunohistochemistry (IHC) results showed that the PNS + BMSCs group could up-regulate the expression of Ki67 protein and cell proliferation. In addition, PNS combined with BMSCs up-regulated the expression of miR-146-5p and down-regulated the expression of IL-1β, IL-6 and TNF-α, IRAK1, TRAF6 and p65 in the NF-κB signaling pathway (*p* < 0.05). Conclusions. PNS combined with bone mesenchymal stem cell transplantation up-regulated miR-146a-5p targeting and binding to IRAK1/TRAF6, inhibiting the activation of NF-κB pathway, which reduced the inflammatory response of DCU and facilitated the skin healing of DCU. Thus, this study provides a theoretical basis and a novel therapeutic option for the treatment of DFU with PNS combined with BMSCs.

**Supplementary Information:**

The online version contains supplementary material available at 10.1186/s13018-022-03467-w.

## Introduction

**Fig. 1:**
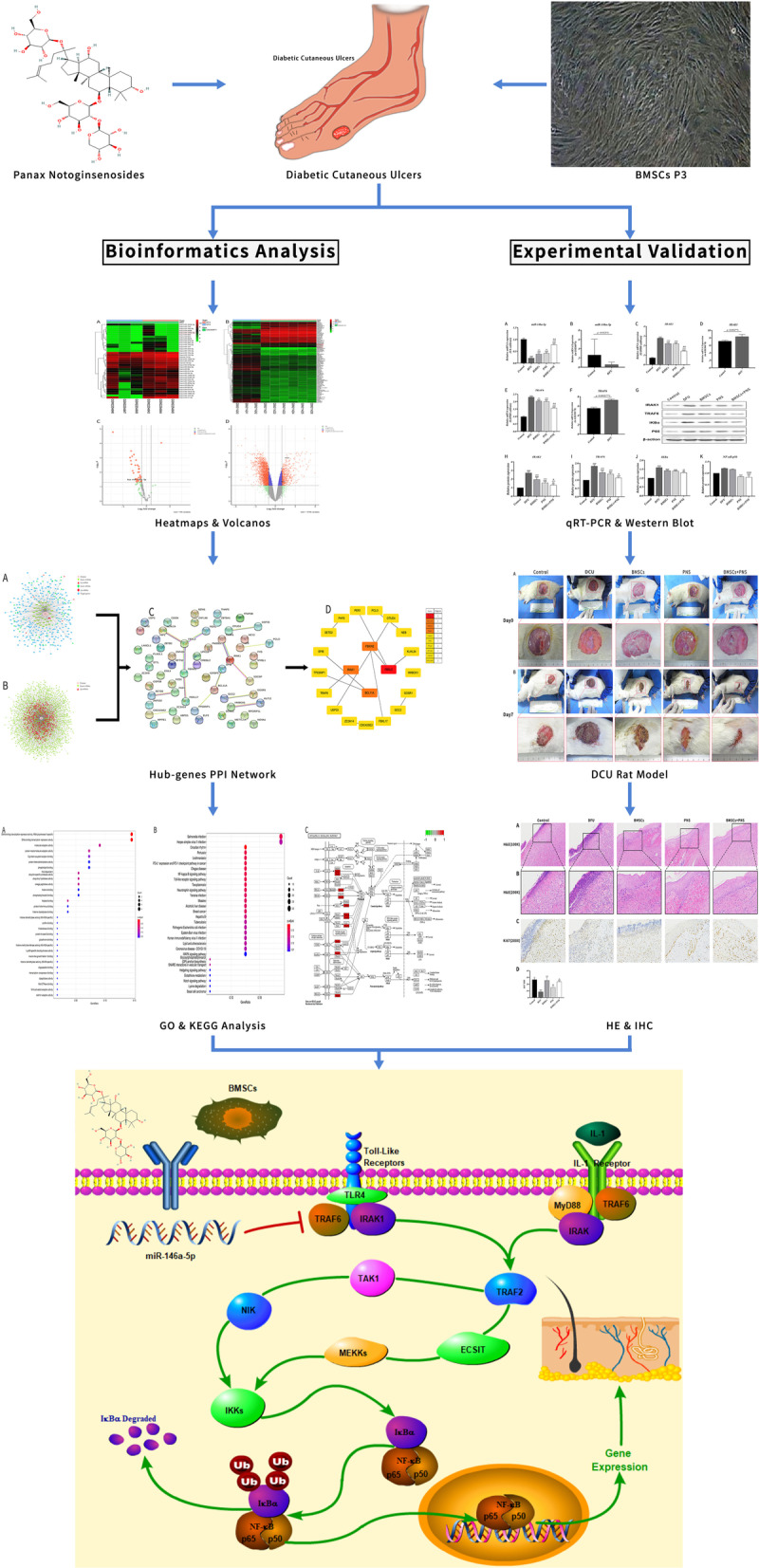
The framework and detailed idea of this study

Diabetes mellitus (DM) refers to a metabolic disease characterized by chronic hyperglycemia due to impaired insulin secretion and/or utilization from multiple causes. Diabetic ulcers (DCU) have been found as a serious complication of diabetes mellitus [1]. Diabetic foot ulcer (DFU), as one of the most severe forms of DCU, is troubling about 6.3% in the world [2]. Diabetic peripheral neuropathy, vascular lesion and inflammation have been considered the pathological mechanism of DCU, which lead to inadequate arterial perfusion and impaired microcirculation. All of these have resulted in the development of skin ulcers and even gangrene in the foot [3]. Existing treatments for DCU consist of anti-infection, hyperbaric oxygen therapy, negative pressure drainage and minimally invasive surgical treatment [4–6], whereas DCU healing is slow, and DU has high recurrence rates [7]. The flow chart of this article is as follows (Fig. 1).

Bone mesenchymal stem cells (BMSCs) refer to a class of adult stem cells of mesodermal origin capable of differentiating into a wide variety of mesenchymal tissues (e.g., bone, cartilage, adipose and bone marrow hematopoietic tissues). BMSCs have been extensively used for treating different diseases. BMSCs have been found with therapeutic effects on DCU by regulating inflammatory mediators, paracrine growth factor-like substances, as well as homing properties [8–10]. Moreover, exosomes derived from BMSC induce the up-regulation of miR-486-3p and suppress oxidative stress and inflammatory responses via the nuclear factor NF-kappa B (NF-κB) signaling pathway [11]. MicroRNAs (miRNAs) refer to a class of non-coding single-stranded RNA molecules down-regulating the expressions of target genes at the post-transcriptional level by pair-binding to their complementary sites [12, 13]. Peng et al. suggested that miR-146a was a possible novel biomarker of inflammation and a potential therapeutic target for the treatment of DCU [14]. Konstantin et al. reported that miRNA-146a down-regulated inflammatory response via the NF-κB signaling pathway [15].

Panax notoginseng (Burkill) F.H.Chen (PN) refers to the dried root and rhizome of PN, a plant of the Wujia family, capable of dispersing blood stasis, stopping bleeding, reducing swelling and fixing pain. It has been known for its efficacy for treating wounds for over 2000 years [16]. Panax notoginseng saponins (PNSs), the first saponin molecule isolated from PN leaves [17], have been found as the major active ingredient of PN [18], which plays a certain role in several biological processes (e.g., reducing inflammatory response, facilitating fibroblast proliferation, as well as promoting angiogenesis) [19, 20]. Furthermore, it has been suggested that under high glucose conditions, PNS induces the M2 phenotypic differentiation of macrophages and activates NF-κB signaling pathway, which plays a role in the polarization of macrophages and reduces the inflammatory response [21].

Accordingly, it is hypothesized that PNS has a certain effect on the NF-κB pathway to reduce inflammatory response. At the same time, many previous studies have found that BMSCs play an anti-inflammatory role in the process of wound healing [22, 23]. Xu demonstrated that miR-146a expression of MSCs was significantly decreased in diabetic wound, and it was related to increase pro-inflammatory target genes IRAK1, TRAF6 and inflammatory genes IL-6 and MIP-2 expression [23]. This study aimed to investigate the molecular basis of PNS combined with BMSCs for treating DCU and its mechanism of action.

## Method

### Analysis of DCU differentially expressed miRNAs and mRNAs expression profiles

The gene microarray data relating to DCU were acquired from Gene Expression Omnibus (GEO) database (https://www.ncbi.nlm.nih.gov/geo/), and the screening conditions consisted of (1) diabetic foot ulcers and (2) human. The microarray data were investigated with the bioconductor R package in R background correction, normalization and expression value calculation, and the limma package was adopted to calculate the differentially expressed miRNAs and mRNAs between the two groups. *P* < 0.05 and ≥ twice the expression change (|log_2_FC| ≥ 1.0) were set as the criteria for screening differentially expressed genes, in which log_2_FC ≥ 1.0 represents the up-regulation of miRNA and mRNA expressions, and log_2_FC ≤ 1.0 represents the down-regulation of miRNA and mRNA expressions. Lastly, the differentially expressed miRNAs and mRNAs of diabetic foot ulcers and non-diabetic foot skin were derived, i.e., diabetic foot ulcer-related differentially expressed miRNAs (DEmiRNAs) and differentially expressed genes (DEGs). The heat map and cluster analysis of the screened DEmiRNAs and DEGs were conducted using heat map package, and *P* value of the differentially processed data was − log_10_ transformed, and − log_10_ (*P* value) was grouped in accordance with log_2_FC (up-regulated miRNAs group, down-regulated miRNAs group, miRNAs group without statistical significance and up-regulated DEGs group, down-regulated DEGs group, as well as DEGs group without statistical significance), and the processed data were plotted in volcano maps with the R. Volcano package. Furthermore, the relative expressions of miRNA-146a-5p, IRAK1, TRAF6 in the microarray data were mined.

### Prediction of miRNA target genes

miRNA target gene was predicted using the DEmiRNAs obtained from the differential analysis in the previous step. DEmiRNAs were entered into the online miRNA target gene prediction websites (e.g., DIANA, MIRANDA, MIRBRIDGE, PICTAR, PITA, RNA22 and TARGETSCAN) for target gene prediction. In addition, the predicted target genes obtained were compared with 2.1 obtained DEGs and then mapped. Furthermore, the ceRNA interaction network model of differentially expressed miRNAs-mRNAs was further built using Cytoscape 3.7.2 software.

### GO and KEGG enrichment analysis

The common potential target genes predicted in the previous step were entered into the DAVID database to select the species as human (Homo sapiens) for Gene Ontology (GO) and Kyoto Encyclopedia of Genes and Genomes (KEGG) signaling pathway analysis. To be specific, GO analysis mainly included cellular component (CC), molecular function (MF) and biological process (BP) of the differentially expressed genes. The target genes were screened at *P* < 0.05, and the biological processes and main signaling pathways of potential target genes were analyzed. Moreover, the Bioconductor-Pathview package was applied in R software (Version R ×64 3.5.1) to build a pathway map relating to DU.

### MTT experiment

The 3rd-generation BMSCs cells were cultured to logarithmic growth phase, the cell suspension concentration was adjusted to 1 × 10^8^/L, inoculated into 96-well culture plates, and PNS was prepared at concentrations of 0, 10, 50, 100 and 200 μg/ml solution and incubated in a 37 °C, volume fraction 5% CO^2^ saturated humidity incubator for 1, 3, 5 and 7 days. The first five parallel wells were incubated with 20 μl MTT (Solarbio, China) per well at 37 °C for 4 h. The wells were centrifuged, the supernatant was discarded, 150 μl dimethyl sulfoxide was added, and the absorbance value of each well was measured by enzyme standardization at 490 nm. The inhibition rate (%) = [(absorbance value of control group − absorbance value of experimental group)/absorbance value of control group] × 100%.

### Construction of DCU rat model

75 8-week-old SPF-grade SD rats, male, weighing 150–200 g, were purchased from the Institute of Animal Science, Chinese Academy of Medical Sciences (Certificate of Conformity No. SCXK (Beijing) 2016-0001). To be specific, 15 rats were randomly selected to be fed with normal chow, and the other 60 rats were fed with high-fat food (HFD) adapted for one months. After the rats adapted to the chow for one month, 60 mg/kg of streptozotocin (STZ, dissolved in 0.1 mmol/L sterile sodium citrate buffer solution at the time of injection, Sigma 3444091) [24] was injected intraperitoneally, and random fasting blood glucose ≥ 16.7 mmol/L was measured by collecting blood from the tail vein at 3 d, 7 d, 14 d and 28 d after injection, which was considered successful modeling. After the rats were anesthetized with pentobarbital sodium, the fur on the back of the rats was removed, and the area was taken up an area of nearly 5 × 5 cm. This area was marked with a 4-cm-diameter circular plastic cap dipped in gentian violet. Under aseptic condition, the skin of the modeling area was cut off to reach the fascia. The wound was covered with medical gauze and fixed with medical paper tape [25]. All animal experimental studies were conducted in accordance with the Animal Ethics Committee of the Chinese Academy of Traditional Chinese Medicine regarding animal research guidelines.

### Primary culture of rat bone marrow mesenchymal stem cells

SPF-grade male SD rats (aged 3–4 weeks) were killed by pentobarbital overdose anesthesia. In addition, the femur was separated. Moreover, the periosteal tissues were removed, soaked in 75% alcohol for 30 s and then washed with PBS containing Penicillin–Streptomycin antibiotics 3 times. The epiphysis at both ends of the bone was cut off, and the bone marrow in the bone stem was washed with α-MEM medium (supplemented with 10% fetal bovine serum) in a 5 ml syringe into a centrifuge tube. The cells were repeatedly blown. The cells were grown on disposable plastic cell culture dishes and then cultured in a cell culture incubator (5%, CO^2^, 37 °C). The unopposed cells were removed through aspiration on day 3 and washed with phosphate buffered saline (PBS) 3 times. Afterward, the apposed cells were continuously cultured. The BMSCs at 3rd passage were taken for later studies. The 3rd-generation BMSCs were rinsed 3 times with PBS, and the walled cells were dislodged by adding 0.05% trypsin at an appropriate amount. Subsequently, the digestion was terminated by adding α-MEM medium at an appropriate amount. The mixed medium was centrifuged at 1000 rpm for 5 min, the supernatant was removed, and the bottom white precipitate was left. Afterward, the BMSCs cell suspension with a cell concentration of 8 × 10^6^ cells/cm^2^ was produced by resuspending with an α-MEM medium at an appropriate amount.

### Grouping and drug administration

The present study was divided into five groups: the control group, 15 rats fed with normal chow with equal amounts of saline to rinse the wounds with the sham operation; the DCU group, 15 rats with diabetic ulcer, equal amounts of saline to rinse the wounds; the BMSCs group, 15 rats with diabetic ulcer were injected by 50 μl (the respective injection site) of BMSCs suspension by intramuscular (6 sites, depth 1 cm, spacing 5 mm); the PNS group, 15 rats with diabetic ulcer were injected Xuesaitong injection 0.3 ml/cm^2^ (Guangxi Wuzhou Pharmaceutical Co) by intramuscular; and the BMSCs + PNS group, 15 rats with diabetic ulcer were injected by BMSCs suspension and Xuesaitong injection, which were the above-mentioned BMSCs group and PNS group administration methods, using at the same time.

All rats were covered with sterile gauze after drug administration. The frequency of administration is once a day. After being treated for 7 days, the rats were anesthetized with pentobarbital, and the blood was collected from the abdominal aorta. Moreover, two copies of granulation tissue (weighing nearly 3 g) were collected from the central point of the wound. One copy of the granulation tissue was fixed by perfusion with 4% neutral paraformaldehyde, paraffin-embedded, and sectioned for pathological analysis. Besides, the other copy was immediately frozen in liquid nitrogen and then stored for the subsequent Western blot (WB) and quantitative real-time polymerase chain reaction (RT-PCR) assays.

### Measurement of ulcer contraction rate

The size of the wound area on the back of the respective rat was measured on the day before (D0) and day 7 (D7) after the intervention. In addition, wound healing was evaluated using the rest percentage of wound surface area (WSA). Wound pictures were captured with a digital camera (DMC-LX5GK; Panasonic, Japan), and the ulcer area was analyzed using Image-Pro Plus 4.5 software.

### HE staining

The ulcerated tissues of the respective group of rats were collected and placed in 4% paraformaldehyde fixed for 24 h. Paraffin was embedded in the tissues. Subsequently, the tissues were sectioned at 5 μm thickness, dewaxed, hydrated, stained with hematoxylin solution-eosin, dehydrated and then sealed with neutral gum, so as to observe the histopathological morphology of the ulcerated tissues of the respective group.

### Immunohistochemical staining

After the tissue sections were dewaxed to water, repaired by antigen and closed, rabbit anti-mouse CD31 polyclonal antibody and rabbit anti-mouse Ki67 monoclonal antibody (Abcam, ab16667) added dropwise to the sections. Subsequently, the sections were incubated for 1 h at 37 °C. The secondary antibody labeled with horseradish peroxidase (HRP) was added and then incubated for 30 min at ambient temperature. Lastly, it was incubated with a DAB kit (ST033, Huijia Biotechnology Co., Ltd., China) for color development, hematoxylin (PT001, Mao Biotech Co., Ltd., China) for light re-staining, dehydration, transparency and blocking. The patches were observed under a microscope, and the percentage of Ki67 positive cells was obtained with ImageJ software to detect Ki67 expression in the respective group of ulcerated tissues. (Ki67 represents a sensitive indicator of cell proliferation.)

### Real-time PCR

The respective group of ulcer granulation tissue was taken. 1 ml of TRIzol reagent was added. The tissue was transferred to EP tube and lysed for 10 min. The 200 μl chloroform was added. The mixture was centrifuged at 4 °C, 12,000 rpm for 15 min, and then, the upper aqueous phase was transferred. The 400 μl isopropanol was added. The solution was centrifuged several times, and the supernatant was discarded. And the precipitate dissolved in 20 μl diethyl pyrocarbonate (DEPC) water. The cDNA was reversely transcribed at 25 °C for 5 min, 50 °C for 15 min, 85 °C for 5 min and 4 °C for 10 min. cDNA was diluted tenfold and then amplified by the real-time quantitative fluorescence PCR reaction system, including 50 °C for 2 min, 95 °C for 10 min, 95 °C for 30 s, 60 °C for 30 s, as well as 40 cycles. The primers were synthesized using GAPDH and U6 as the internal primers. GAPDH and U6 served as the internal reference genes, and the primer sequences are listed (Additional file 1: Table S1).

### Western blot

100 mg of sarcomeric tissue was taken in a Petri dish and cut into small pieces of nearly 3 mm × 3 mm. 0.5–1 mL of cold lysis buffer (containing 5 μl of phosphatase inhibitor, 1 μl of protease inhibitor and 5 μl of 100 mM phenylmethylsulfonyl fluoride (PMSF) was added, and the tissue was ground into homogenate. The tissue homogenate was transferred to a 1.5 mL pre-chilled centrifuge tube and centrifuged at 12,000* g* for 5 min at 4 °C. Moreover, the supernatant was taken as the total tissue protein extract, and the protein concentration was examined. The extracted proteins were added with 5 × sodium dodecyl sulfate (SDS) loading buffer. The mixture was placed into boiling water to denature the proteins and centrifuged at 12,000 r/min for 5 min. The proteins were separated through SDS–polyacrylamide gel electrophoresis (PAGE) electrophoresis and then transferred to polyvinylidene fluoride membranes (PVDF membranes). Afterward, the proteins were closed with 5% skim milk powder at ambient temperature for 1 h. The PVDF membranes subjected to β-actin (Affinity, 1:3000), trafficking kinesin-binding protein 1 (IRAK1) (Affinity,1:1000), TNF receptor-associated factor 6 (TRAF6) (Affinity, 1:1000), NF-kappa-B inhibitor alpha (IKBα) (Affinity, 1:1000), NF-κB p65 (Affinity, 1:1000), IL-1 (Abcam, 1:1000), IL-6 (Abcam,1:1000), TNF-α (Abcam, 1:1000) primary antibodies were incubated overnight at 4 °C and rinsed 3 times with TBST. Furthermore, the PVDF membranes were incubated in HRP-labeled rabbit secondary antibody solution diluted at 1:5000 for l h. The protein bands were collected by adding enhanced chemiluminescence (ECL) luminescent solution at an appropriate amount and developed with ImageJ software to calculate the grayscale values of the protein bands. On that basis, the relative expression of the respective group of proteins was measured.

### Statistical analysis

The experimental data were statistically analyzed with SPSS 25.0 software, and the data are expressed as mean ± standard deviation (${\overline{\text{x}}}$ ± s). The data displaying normal distribution were analyzed using one-way ANOVA, and the two-way comparison of data with the same variance was drawn through the LSD test with *P* < 0.05 as the test standard.

## Results

### DCU-related differentially expressed miRNAs and mRNAs expression profiles

**Fig. 2:**
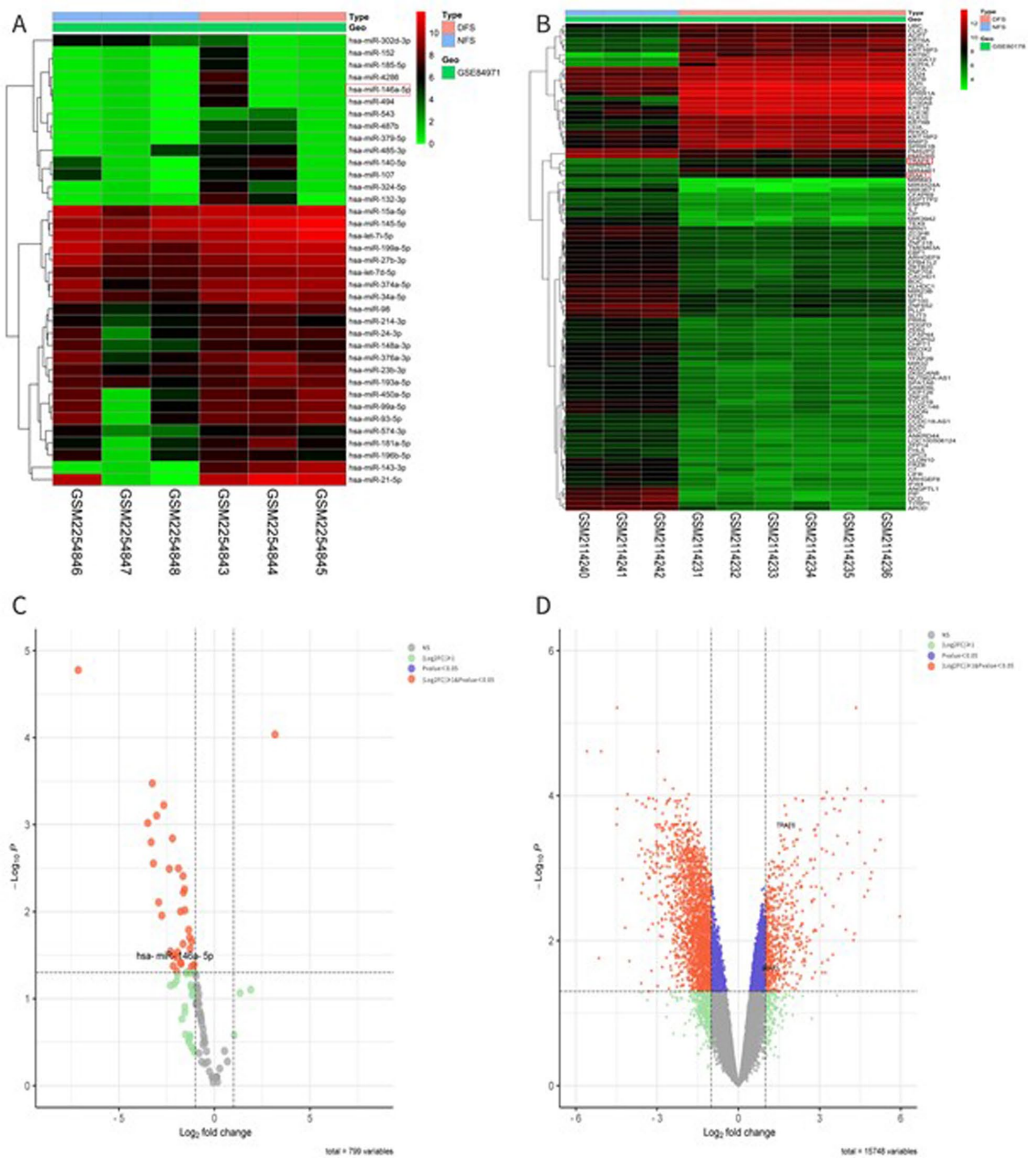
Bioinformatics analysis of GSE84971 and GSE80178. **A** Heat map of total 37 DEmiRNAs in GSE84971; **B** Heat map of top 100 DEGs in GSE80178. (Tissue samples are presented as columns; individual genes are presented as rows. Red indicates up-regulated genes and green indicates down-regulated genes in patients with DFS. The patients in the top rows in blue indicate columns were normals; the patients in the top rows in pink indicate columns were patients with DFS.); **C** The volcano plot of total 37 DEmiRNAs in GSE84971; **D** The volcano plot of total 2628 DEGs in GSE80178. (The vertical black lines correspond to Log_2_FC up and down, and the horizontal black line represents the *P* value of 0.05. The red point represents the differentially expressed genes with statistical significance.) (DFS, diabetic foot skin; NFS, non-diabetic foot skin)

The study data were acquired from miRNA microarray dataset GSE84971 of 3-foot skin samples from patients with DCU and 3-foot skin samples from non-diabetic patients. Moreover, the DEmiRNAs and DEGs in GSE84971 and GSE80178 were plotted according to the *P* value. In accordance with the *P* value < 0.05 and expression change of higher than or equal to twofold (|log_2_ FC| ≥ 1.0) as the criteria for screening differentially expressed genes, 37 differentially expressed miRNAs (1 up-regulated miRNA and 36 down-regulated miRNAs) were screened in the GSE84971 dataset, and 2628 differentially expressed genes (579 up-regulated genes and 2049 down-regulated genes) were screened in the GSE84971 and GSE80178 datasets. Moreover, the DEmiRNAs and DEGs in GSE84971 and GSE80178 were plotted in accordance with the *P* value significance (Fig. 2A, B), where red and green represent the up-regulated gene expression and the down-regulated gene expression, respectively. The processed data were imported into GraphPad Prism 8.0 to plot the volcanoes (Fig. 2C, D).

### miR-146a-5p target gene prediction

**Fig. 3:**
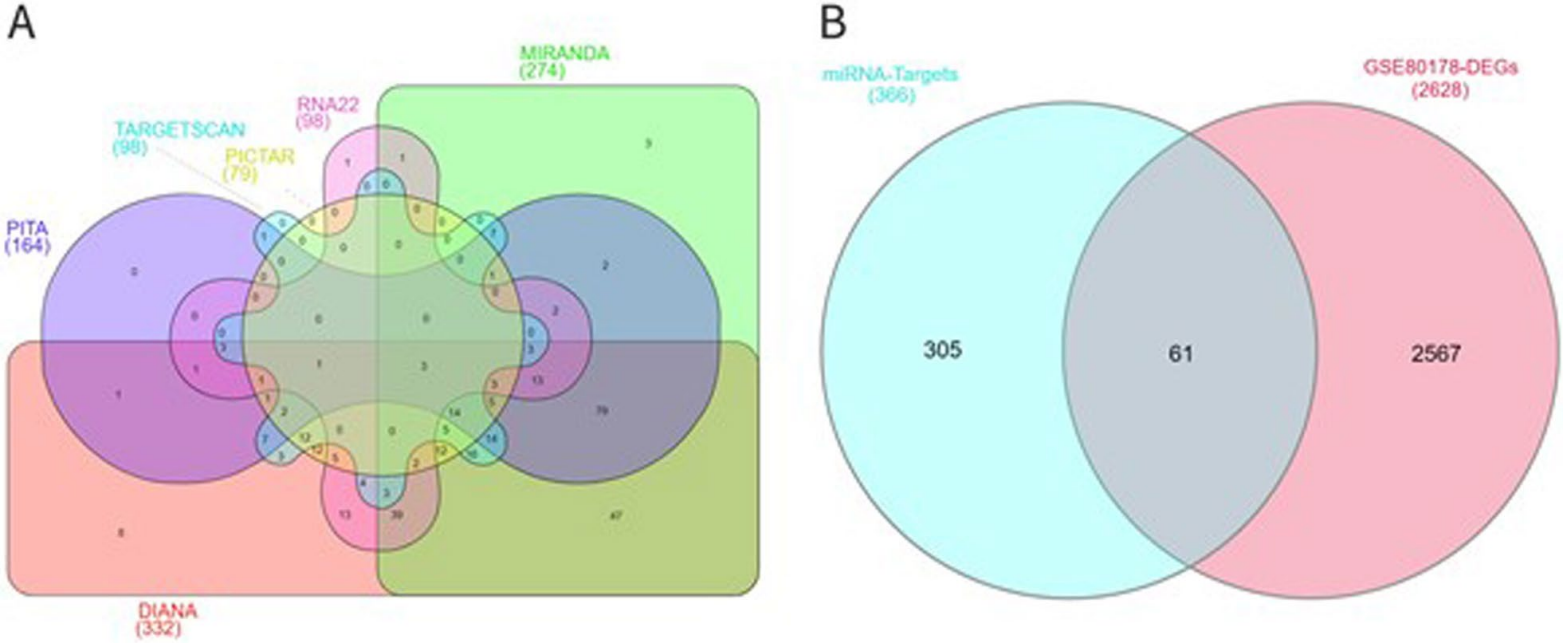
Prediction of miR-146a-5p target genes. **A** Venn diagram of predicted target genes of hsa-miR-146a-5p; **B** Venn diagram between predicted-related targets of hsa-miR-146a-5p and the DEGs of GSE80178

miR-146a-5p obtained from the differential expression analysis was used for target gene prediction and input into the online miRNA target gene prediction websites (e.g., DIANA, MIRANDA, MIRBRIDGE, PICTAR, PITA, RNA22 and TARGETSCAN) for target gene prediction (Fig. 3A). Moreover, 366 potential target genes were obtained, binding to miR-146a-5p 366 potential target genes that bind to miR-146a-5p were obtained. In comparison with DEGs obtained by the differential analysis of pre-chip data, 61 common target genes were finally obtained. As compared with target genes with the binding relationship with miR-146a-5p verified by literature experiments, the results are listed in Table 1, which consisted of TRAF6, IRAK1, BRCA1, PBLD, BRCA2 and others, common target genes Venn diagram (Fig. 3B).

**Table 1:** miR-146a-5p potential target genes

Target gene	Validation	DIANA	MIRANDA	MIRBRIDGE	PICTAR	PITA	RNA22	TARGETSCAN	Total
TRAF6	√	√	√	√	√	√	√	√	8
IRAK1	√	√	√	√	√	√	–	√	7
KLF7	–	√	√	√	√	√	√	√	7
DLGAP1	–	√	√	√	√	√	–	√	6
ELF2	–	√	√	√	√	√	√	–	6
NOVA1	–	√	√	√	√	√	–	√	6
PSMD3	–	√	√	√	√	√	–	√	6
RUNX1T1	–	√	√	√	√	√	–	√	6

### DCU core target genes

**Fig. 4:**
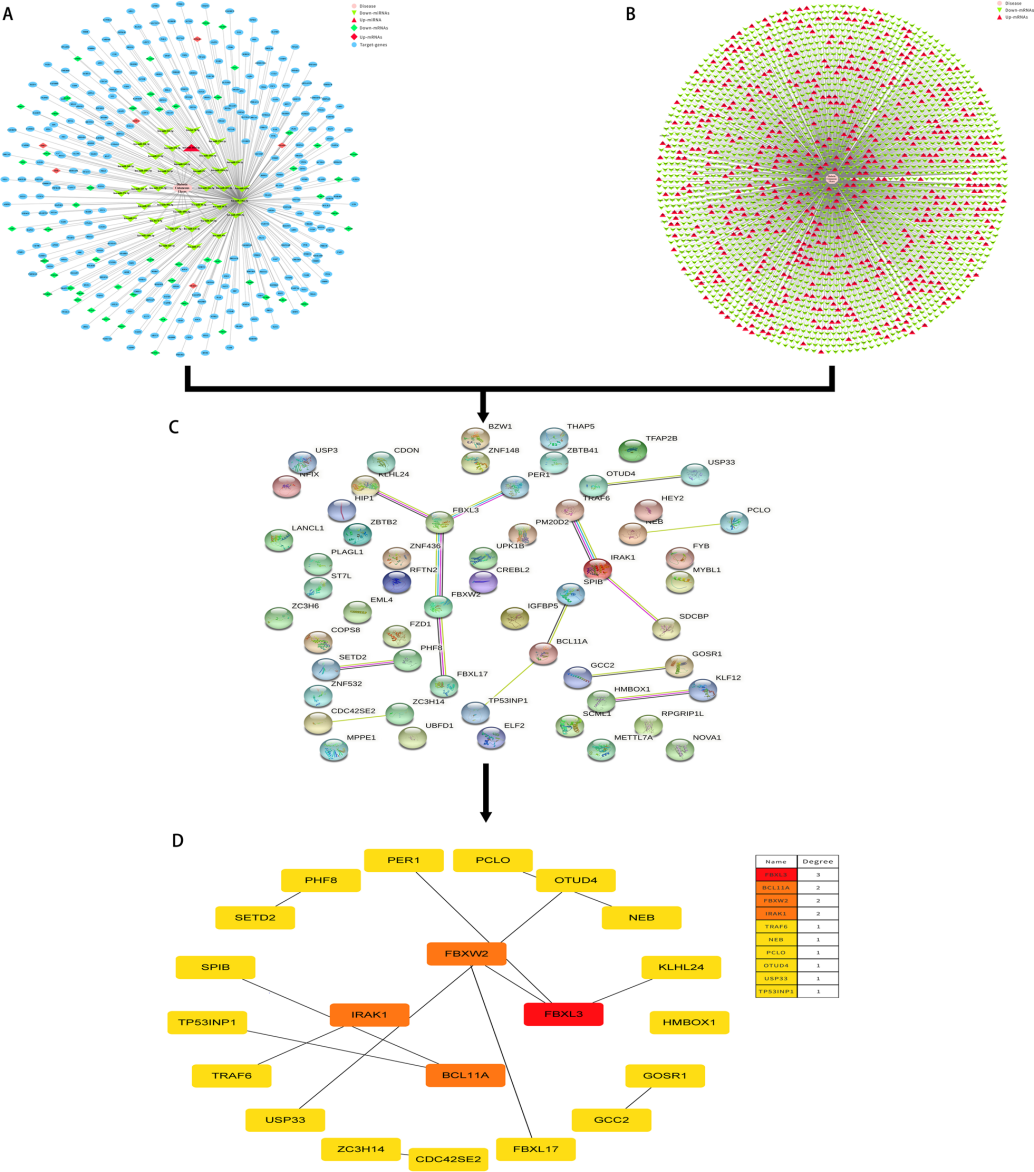
Process of topological screening for the PPI network. **A** PPI network of DEmiRNAs-related targets. **B** PPI network of DCU differentially expressed genes. **C** PPI network of key targets of merging mapping. **D** PPI network of key genes by cytoHubba

The networks of disease DEmiRNAs and disease DEGs were built using Cytoscape 3.7.2 software (Fig. 4A, B), and the two networks were combined to obtain the DCU-DEmiRNAs-DEGs network model, and the 61 common target genes obtained in 3.2 were imported into STRING online analysis website (https://string-db.org/). “Medium confidence > 0.400” was set in the minimum interaction score, and the protein–protein interaction results and network diagram were exported (Fig. 4C). Further, the hub genes were screened using the cytoHubba plug-in in the Cytoscape software, and the potential core target genes were derived by the Degree algorithm (Fig. 4D).

### DCU biological function (GO) and core pathway (KEGG)

**Fig. 5:**
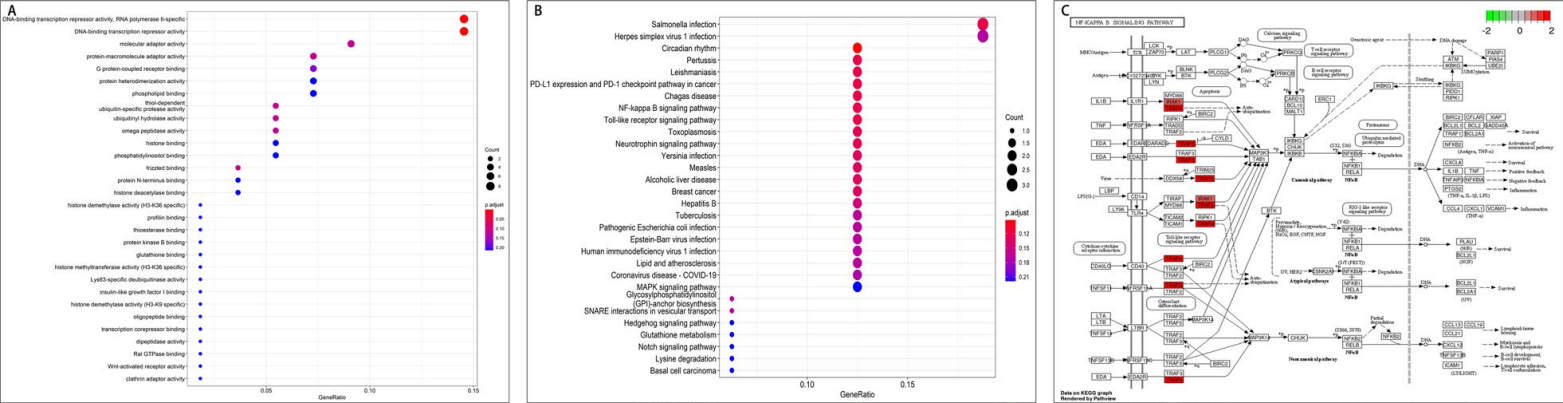
GO and KEGG enrichment analyses of 61 candidate targets. **A** The Bubble chart of the top 30 terms extracted according to the *p*.adjust value based on GO enrichment analysis. **B** The Bubble chart of the top 30 pathways extracted according to the *p.*adjust value based on KEGG enrichment analysis. **C** NF-κB signaling pathway map of DCU targets. (Red nodes represent the most potential targets, arrows represent the activation effect, T arrows represent the inhibition effect, and segments show the activation effect or inhibition effect.)

GO and KEGG pathway enrichment analysis was conducted on 61 core targets of DCU using the Bioconductor package and cluster Profiler package in R language. As indicated by the GO analysis, they were primarily enriched in DNA-binding transcription repressor activity, RNA polymerase II-specific, molecular adaptor activity, etc. (Fig. 5A). As revealed by the KEGG pathway enrichment analysis, they primarily focused on the NF-κB signaling pathway, TLR signaling pathway, as well as mitogen-activated protein kinase (MAPK) signaling pathway (Fig. 5B). Moreover, the pathview package was adopted to show the DCU-related NF-κB signaling pathway map (Fig. 5C).

### Effect of PNS on the BMSCs proliferation

**Fig. 6:**
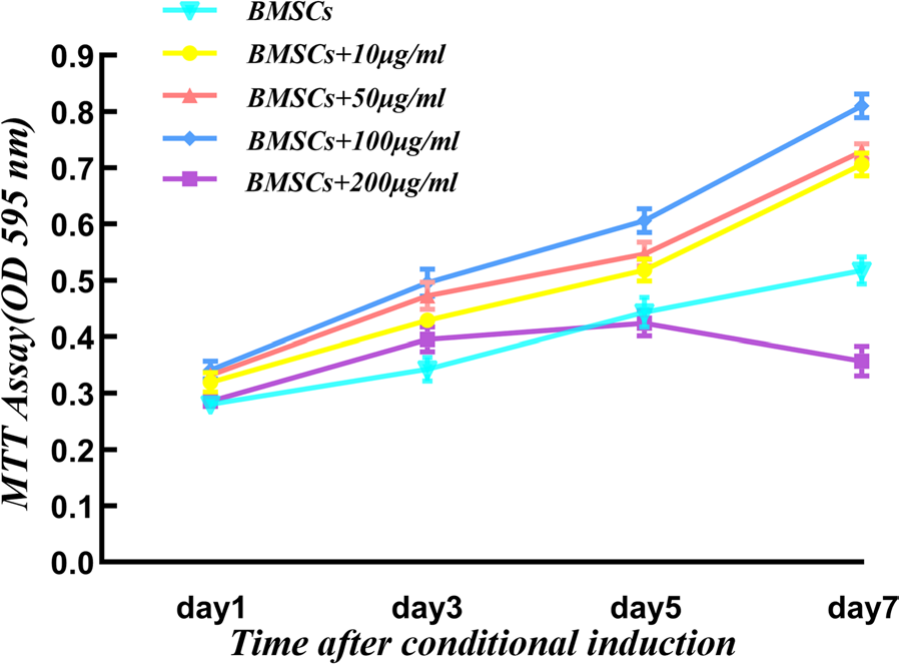
Proliferative effects of PNS on BMSCs. These represent the mean ± SD in fourth times independent experiments (*n* = 4). *P < 0.05 versus BMSCs group, **P < 0.01 versus BMSCs group, ***P < 0.001 versus BMSCs group; #*P* < 0.05 versus 10 μg/ml PNS group; &*P* < 0.05 versus 50 μg/ml PNS group

The proliferation of BMSCs exposed to concentrations of 0–200 μg/ml of PNS was monitored over 7 days of culture. Day 1 was the latent period for cell proliferation, and cells started to proliferate from day 3. Over 7 days, PNS significantly promoted the proliferation of BMSCs at 10, 50 and 100 μg/ml final concentrations. Meanwhile, the cell proliferation activity of the 200 μg/ml PNS group decreased rapidly after 5 days of culture, indicating that PNS at this (or higher) concentration had an inhibitory effect on cell growth (Fig. 6). 10, 50 and 100 μg/ml PNS had no significant toxic effects on BMSCs cells, and 100 μg/ml PNS had the most significant effect on BMSCs cell proliferation.

### Changes in wound healing in DCU rats

**Fig. 7:**
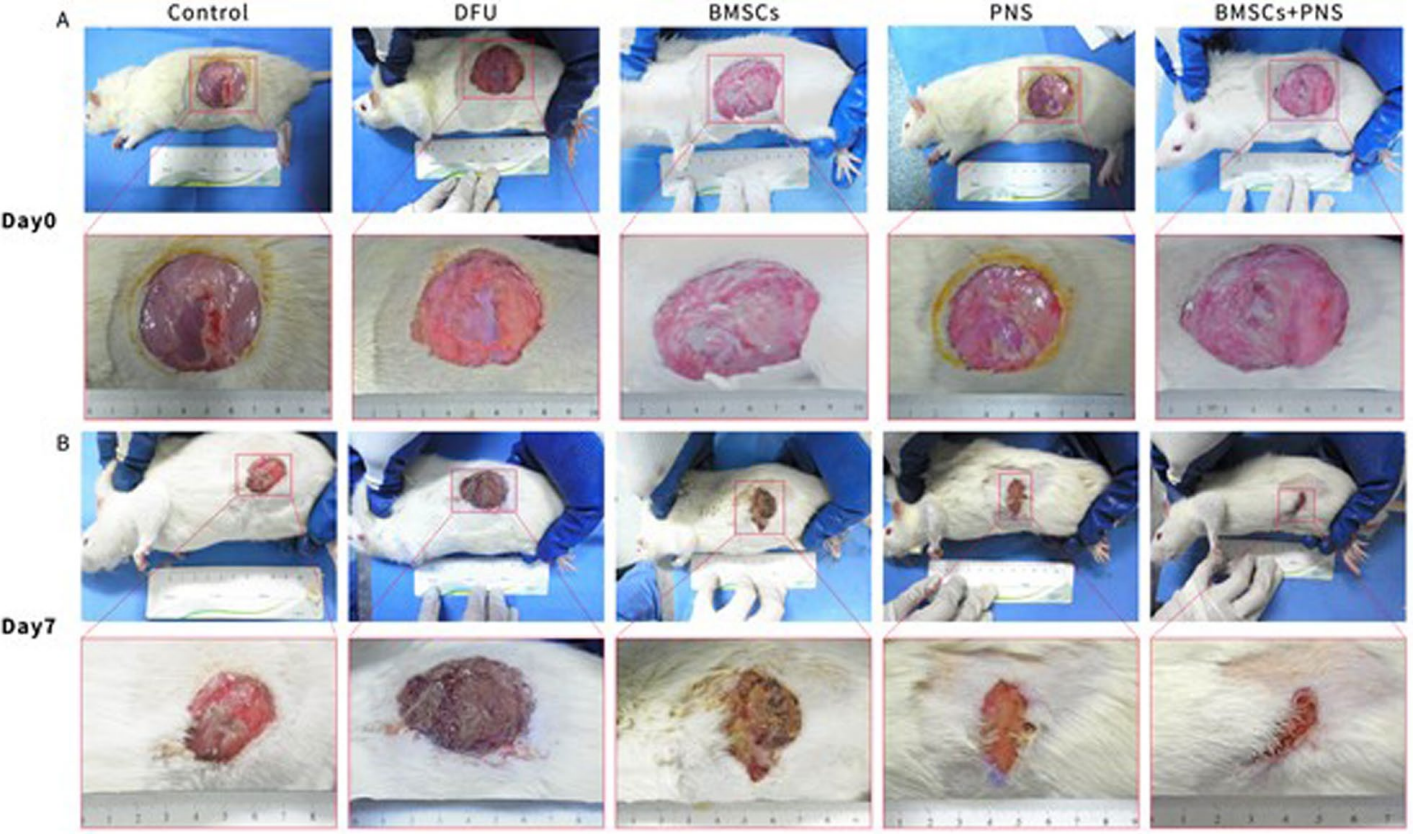
Wound changes of DCU rat model. **A** Wounds on the day of modeling in different groups of DCU rats. **B** Wounds on the 7th day after intervention in different groups of DCU rats

**Fig. 8:**
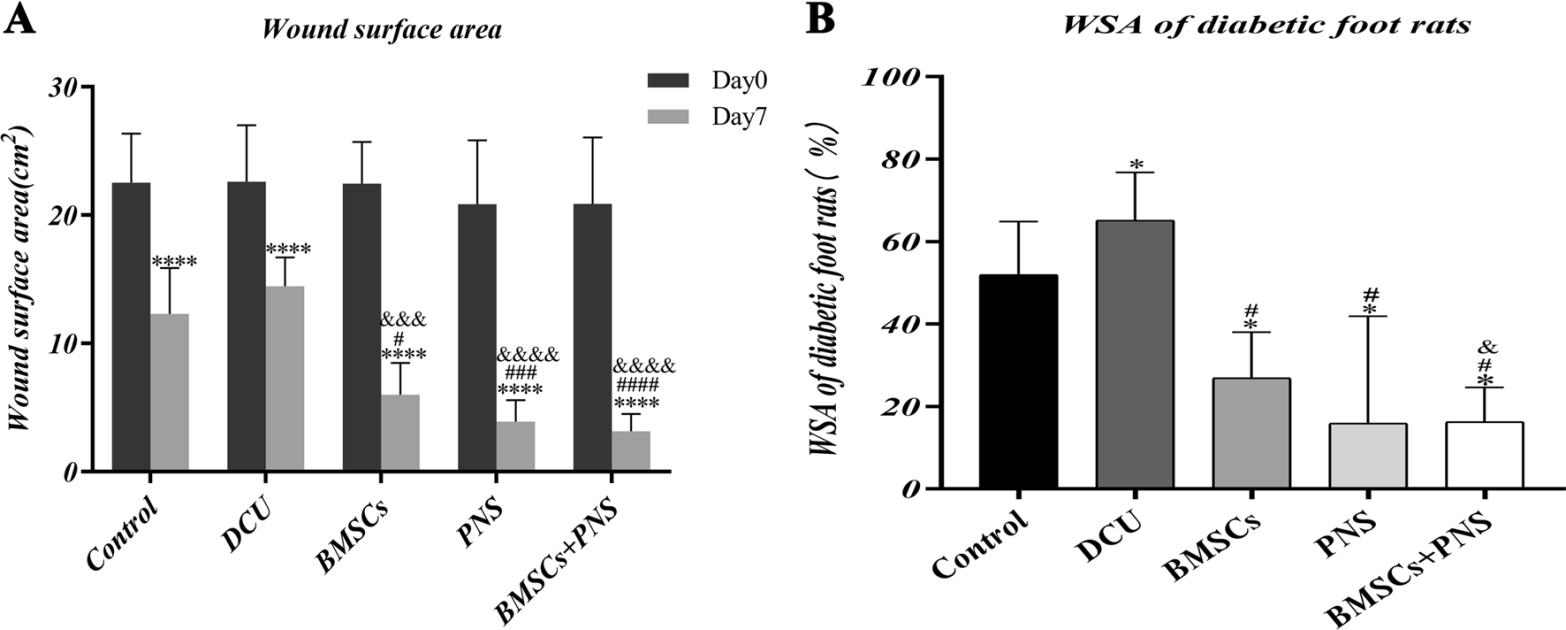
Changes in wound healing in DCU rats. **A** Wound surface area of diabetic foot rats before and after treatment. (*P < 0.05 vs day 0, #*P* < 0.05 vs control group day 7, &*P* < 0.05 vs DCU group day 7). **B** WSA of diabetic foot rats. (*P < 0.05 vs control, #*P* < 0.05 vs DCU, &*P* < 0.05 vs PNS)

After 7 d of intervention, the traumatic surface of the respective group healed to different degrees. It was found that the traumatic surface of the control group was crusted off, and the wound was shrunken and red. The traumatic surface of the DCU group showed a yellow–brown crust with a small amount of thin purulent secretion. The traumatic surface of the BMSCs group showed a red-brown crust with scattered flakes as well as a small amount of clear and thin exudate. The traumatic surface of the PNS group was red and alive, and the traumatic surface was effectively healed with hair growth around the trauma. The BMSCs + PNS group showed the largest wound healing area, the most skin growth at the wound edge, as well as obvious granulation tissue filling of the wound (Fig. 7A, B). There was no statistically significant difference in the wound area of the respective group before treatment. Moreover, after 7 d of intervention, the wound area of the respective group was significantly reduced as compared with that at day 0, and the difference had statistical significance. Furthermore, the wound areas of the BMSCs group, the PNS group and the BMSCs + PNS group were significantly reduced compared with the DCU group on day 7 (Fig. 8A).

The remaining percentage of wound surface area (WSA) was adopted to assess wound healing, and the default D0 was 100% WSA. In comparison with the DCU group, the BMSCs group, the PNS group and the BMSCs + PNS group had significantly reduced wound WSA, and the differences had statistical significance, which revealed that BMSCs transplantation, PNS injection and MSCs combined with PNS could promote skin ulcer healing in the diabetic model rats, and the BMSCs + PNS group showed the smallest remaining surface area of the diabetic rat wounds, thus significantly facilitating wound healing (Fig. 8B).

### Effect of BMSCs combined with PNS on skin ulcers of DCU rats

**Fig. 9:**
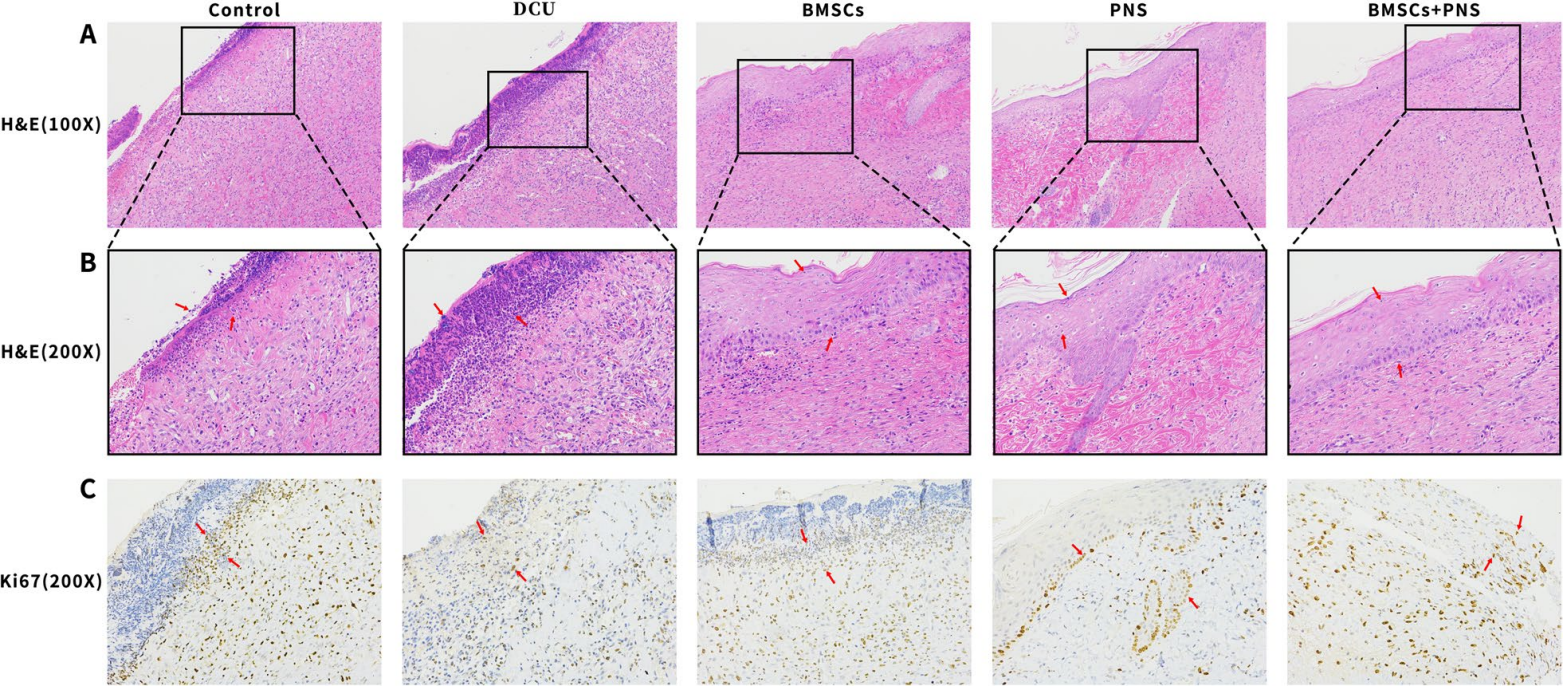
HE staining and IHC of rats granulation tissue in different groups. **A** HE-stained sections at × 100 of skin granulation tissue in different groups of DCU rats. **B** HE-stained sections at × 200 of skin granulation tissue in different groups of DCU rats. **C** Ki67 IHC-stained sections at × 200 of skin granulation tissue of different groups of DCU rats. (Note: Red arrows represent neutrophils in HE and red arrows represent Ki67 in IHC.)

As indicated by the HE-stained granulation tissue sections, the control group had a regenerated epidermis with a small number of fibroblasts and collagen, and numerous capillaries in the dermis. In the DCU group, there was thinning skin, disorganized and few epidermal cells, scattered fibroblasts, few neovascularizations, less lumen, as well as a large infiltration of inflammatory cells. In the BMSCs group, there was a new epidermis, more fibroblasts, thin dermis, as well as a considerable number of inflammatory cells. In the PNS group, new epidermis appeared, more fibroblasts, collagen fibers were arranged neatly and regularly, the dermis was thickened, and the inflammatory reaction zone of the trauma receded. As compared with the DCU group, the number of inflammatory cells decreased (Fig. 9A, B). The above results revealed that BMSCs and PNS facilitated the formation of new epidermis and deposition of collagen fibers in DCU granulation tissue and reduced inflammatory cell infiltration, thus promoting wound healing in DCU rats. Furthermore, BMSCs combined with PNS could increase epidermal tissue and significantly reduce inflammatory cell infiltration in comparison with the result of the application of BMSCs and PNS alone.

### BMSCs combined with PNS increased Ki67 to accelerate DCU rats healing

**Fig. 10:**
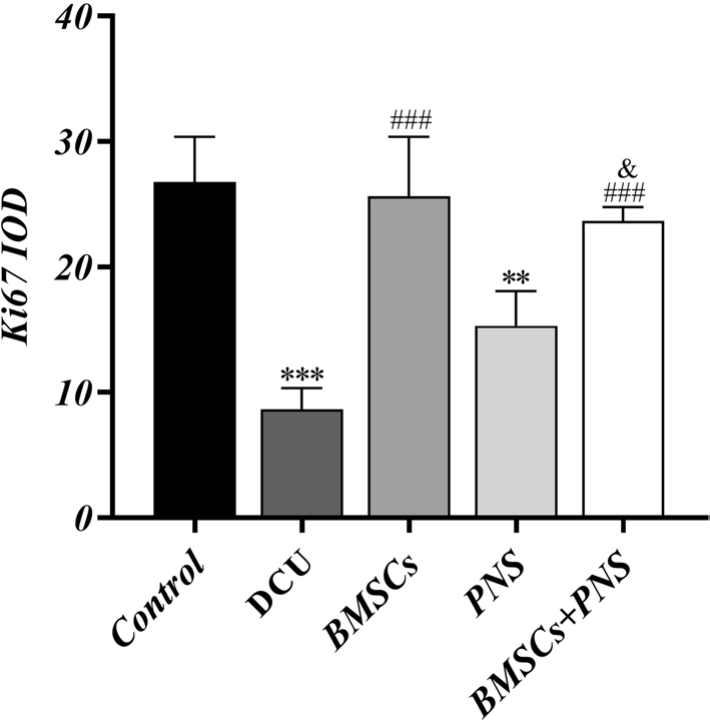
The average optical density of Ki67 in each group. (**: vs control group, *p* < 0.01; ***: vs control group, *p* < 0.001; ###: vs DCU group, *p* < 0.001; &: vs PNS group, *p* < 0.05.)

As revealed by the immunohistochemistry (IHC) results, the number of Ki67+-expressing cells significantly increased in the BMSCs group, the PNS group and the BMSCs + PNS group compared with that of the DCU group, whereas there was no significant difference in the number of Ki67+-expressing cells between the BMSCs + PNS group and the BMSCs and PNS groups (Figs. 9C, 10). The above results suggested that both BMSCs and PNS could promote the increase of the number of Ki67 protein + expressing cells and facilitate the proliferation of ulcer tissue cells, thus accelerating the repair of DCU ulcer.

### BMSCs combined with PNS facilitated the up-regulation of miR-146a-5p expression relative to the DCU group

**Fig. 11:**
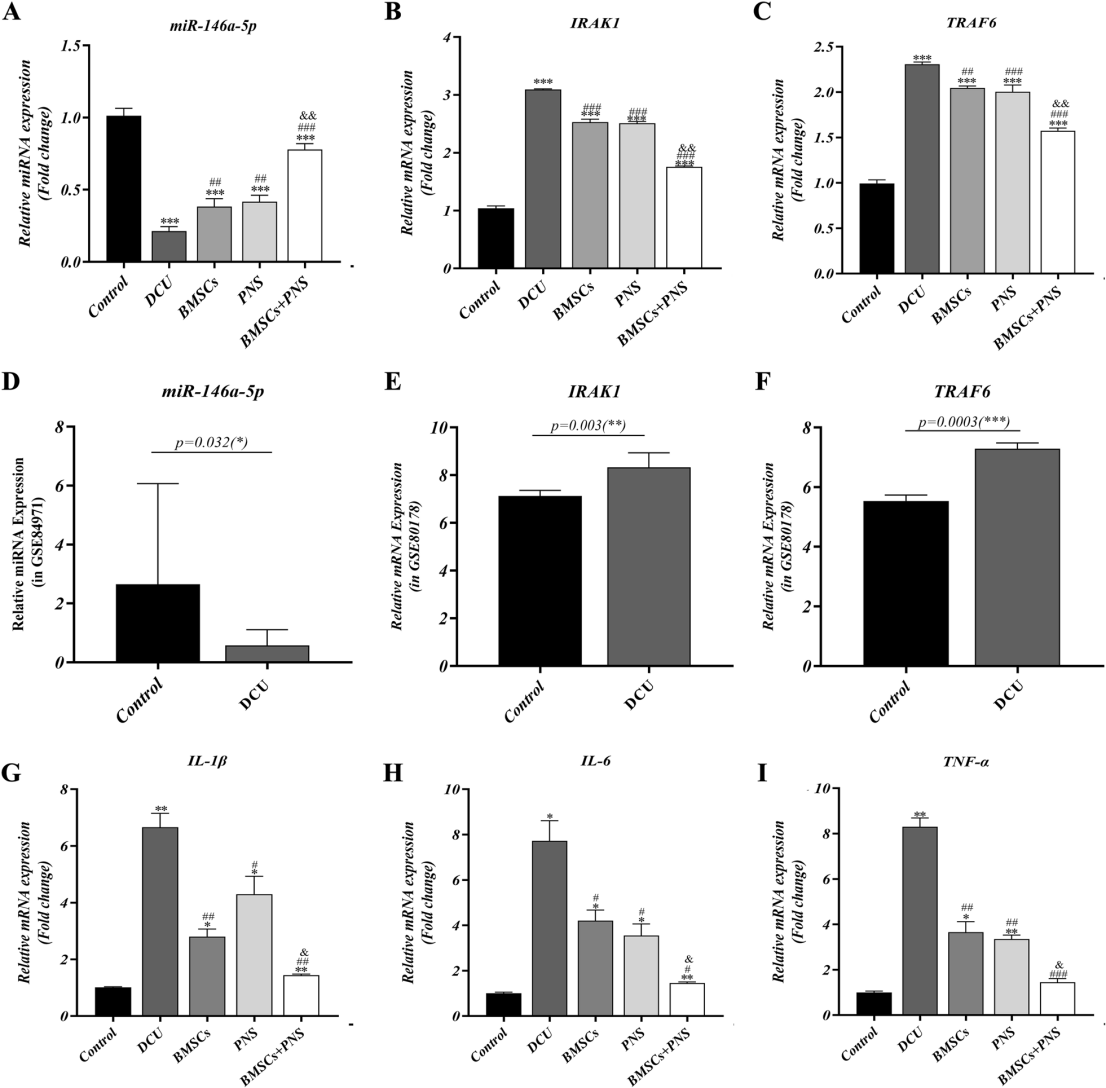
Representative PCR analyses of miR-146a-5p, IRAK1, TRAF6 and related inflammatory factor expression in different groups. **A**–**C** Relative expression of miR-146a-5p, IRAK1, TRAF6 in granulation tissues of DCU rats in different groups. **D**–**F** Relative expression of miR-146a-5p, IRAK1, TRAF6 in human DCU tissue based on GSE84971 database. **G**–**I** Relative expression of IL-1β, IL-6, TNF-α in granulation tissues of DCU rats in different groups. (*: vs control, *p* < 0.05; **: vs control, *p* < 0.01; ***: vs control, *p* < 0.001; ##: vs DCU, *p* < 0.01; ###: vs DCU, *p* < 0.001; &: vs PNS/BMSCs, *p* < 0.05; &&: vs PNS/BMSCs, *p* < 0.01; &&&: vs PNS/BMSCs, *p* < 0.001)

Compared with the control group, the expression of miR-146a-5p in the DCU group, the BMSCs, the PNS group and the BMSCs + PNS group was down-regulated to different degrees, consistent with the change of miR-146a-5p expression in the previous 3.1 microarray data (Fig. 11A, D). In comparison with the DCU group, the BMSCs, PNS group and BMSCs + PNS group showed that the expression of miR-146a-5p was up-regulated to different degrees, and the expression of miR-146a-5p increased most significantly in the BMSCs + PNS group. As revealed by the above results, the expression of miR-146a-5p was down-regulated in ulcerated tissues in DCU, while BMSCs combined with PNS significantly up-regulated the expression of miR-146a-5p in ulcerated tissues.

### BMSCs combined with PNS inhibited the development of DCU inflammation by inhibiting the NF-κB signaling pathway and reducing the expressions of IRAK1 and TRAF6

**Fig. 12:**
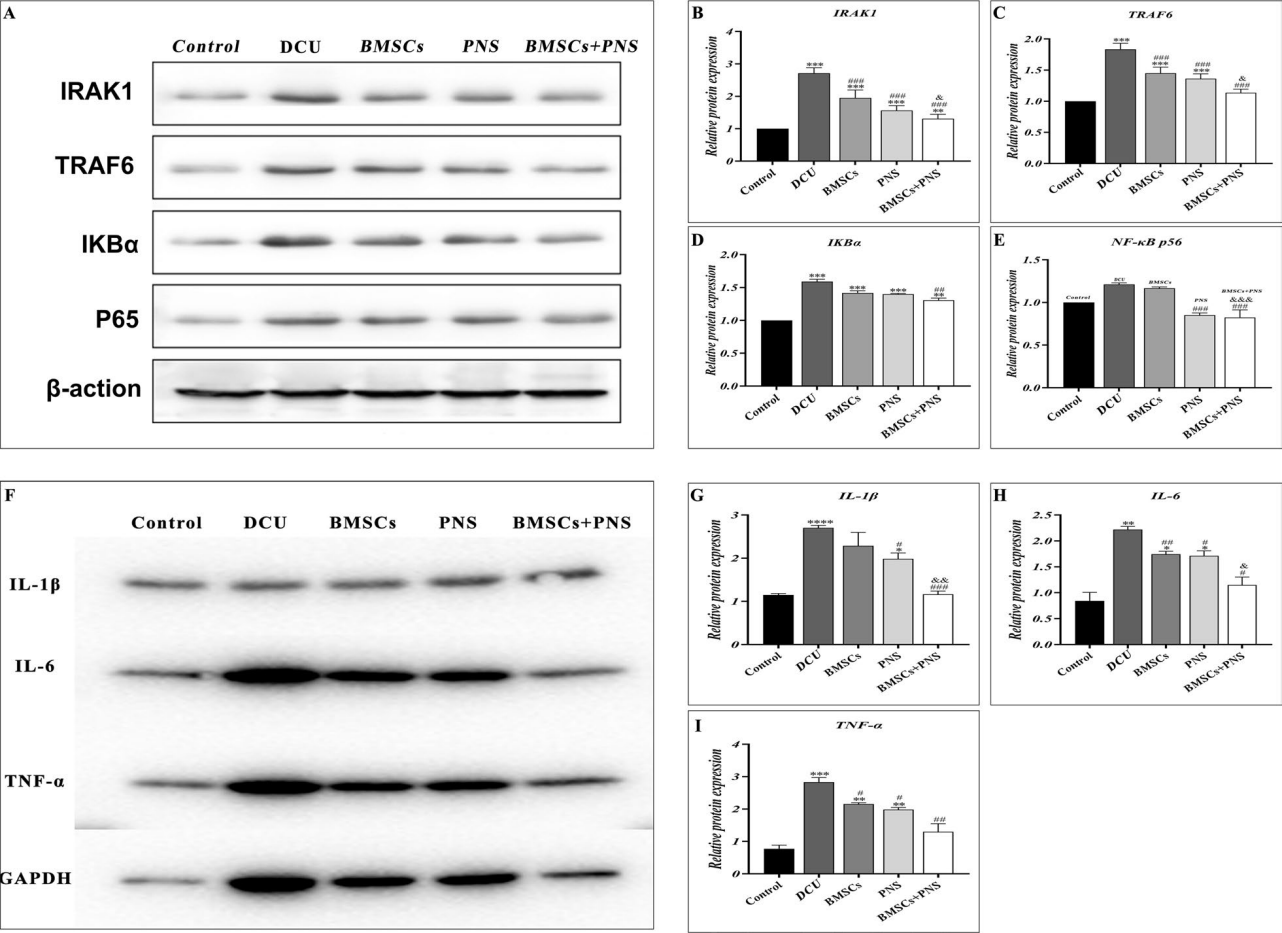
Representative Western blot IRAK1, TRAF6, IKBα, NF-κB p65 and related inflammatory factor expression in different groups. **A** Protein band map of in related pathway of DCU rats in different groups. **B**–**E** Relative quantification of IRAK1, TRAF6, IKBα, NF-κB p65 protein in granulation tissues of DCU rats in different groups. **F** Protein band map of inflammatory factors of DCU rats in different groups. **G**–**I** Relative quantification of IL-1β, IL-6, TNF-α protein in granulation tissues of DCU rats in different groups

The expressions of IRAK1 and TRAF6 were detected through PCR and WB. As indicated by the results, the expressions of IRAK1, TRAF6, IL-1β, IL-6 and TNF-α were significantly higher in the DCU group, BMSCs, PNS group and BMSCs + PNS group compared with control group, consistent with the changes of the expressions of IRAK1, TRAF6, IL-1β, IL-6 and TNF-α in the previous 3.1 microarray data (Figs. 11B, C, E–I, 12G–I). Compared with the DCU group, the expressions of IRAK1, TRAF6, IL-1β, IL-6 and TNF-α in the BMSCs, the PNS group and the BMSCs + PNS group decreased to different degrees, with the most significant decrease in IRAK1, TRAF6, IL-1β, IL-6 and TNF-α in the BMSCs + PNS group. Besides, compared with control group, the expression of p65 in BMSCs group and DCU group had no statistical difference. And the expression changes of NF-κB signaling pathway star molecules IKBα and P65 were consistent with IRAK1 and TRAF6 (Fig. 12B–E). As revealed by the above results, BMSCs + PNS could down-regulate the expressions of IRAK1 and TRAF6 in DCU by inhibiting the activation of the NF-κB signaling pathway, which could suppress inflammation.

## Discussion

DCU has been found as the most common complication of type 2 diabetes mellitus characterized by high disability and mortality [26]. The pathogenesis of DCU is complex, and chronic inflammation has been reported as an important cause of persistent DCU [27]. There seen numerous DCU treatments available, whereas none of them are effective and inexpensive, which imposes more economic burden [28]. Over the past few years, BMSCs transplantation through miRNA-NF-κB has been found to facilitate wound repair [29, 30]. It has also been reported that PNS activation of NF-κB signaling pathway exerts good anti-inflammatory effects [31], while whether PNS topical application combined with BMSCs transplantation can facilitate DCU wound healing has been rarely studied. It was found through bioinformatics analysis and experimental validation that PNS combined with BMSCs transplantation could up-regulate miR-146a-5p, inhibit IRAK1/TRAF6/NF-κB p65 signaling pathway and accelerate DCU wound repair.

Previously, we found that miR-146a-5p was lowly expressed in DCU through bioinformatics analysis, and IRAK1 and TRAF6 were binding target genes of miR-146a-5p [32]. Besides, IRAK1 and TRAF6 were up-regulated in DCU. The enrichment of 61 target genes in DCU revealed that they were primarily involved in steroids. Furthermore, the enrichment analysis of the KEGG pathway suggested that the core targets were largely involved in the NF-κB signaling pathway, the TLR signaling pathway and the MAPK signaling. Furthermore, the PPI network suggested that core targets (e.g., IRAK1, BCL11A, FBXW2, FBXL3 and TRAF6) were closely correlated with wound repair and anti-inflammatory response, while playing an important role in the therapeutic mechanism of PNS combined with BMSCs for DCU. Next, a series of experiments were performed to verify our predictions and analysis.

PNS is also the main component of Chinese herbal medicine, which has the effects of vasodilation, reducing myocardial oxygen consumption, inhibiting platelet agglutination, extending clotting time and scavenging free radicals [33, 34], while exerting pharmacological effects (e.g., anti-inflammatory and antioxidant damage) [35, 36]. In a rat model of diabetic foot ulcer, intramuscular injection of PNS combined with BMSCs transplanted on the ulcer showed that either BMSCs or PNS accelerated DCU healing during wound healing, consistent with previous findings [37, 38], whereas BMSCs combined with PNS DCU rats healed were the fastest. As indicated by the HE staining results in this study, PNS reduced inflammatory cells in ulcerated tissues [39, 40], while BMSCs also reduced inflammatory response and facilitated epidermal regeneration [41]. Besides, PNS combined with BMSCs more significantly reduced inflammatory cell infiltration and increased the number of epidermal cells. Furthermore, IHC results suggested that both BMSCs and PNS facilitated cell proliferation in ulcerated tissue, whereas the combined effect was not stronger than that of BMSCs or PNS alone in promoting Ki67 expression. The above results revealed that either PNS or BMSCs could inhibit the inflammatory response of DCU and facilitate wound repair, while PNS combined with BMSCs was found to be more effective.

To further elucidate the molecular mechanisms underlying the anti-inflammatory effects of PNS combined with BMSCs in DCU, the expressions of miR-146a-5p, IRAK1, TRAF6 mRNA and inflammatory genes correlated with DCU were further investigated. miR-146a, a key regulatory molecule in the inflammatory response, induced different pro-inflammatory stimulators, including interleukin-1β (IL-1β) and tumor necrosis factor-α (TNF-α), among others [42]. Moreover, miRNA-146a has also been reported to inhibit IRAK1 and TRAF6 by targeting and inhibiting them [43, 44]. In this study, the expression of miR-146a-5p was down-regulated, and the expressions of IRAK1 and TRAF6 were up-regulated in DCU tissues. In contrast, PNS combined with BMSCs significantly up-regulated miR-146a-5p expression and decreased the expressions of RAK1 and TRAF6. Existing studies revealed that PNS is correlated with several miRNAs [45, 46], and this study has been the first to find that PNS facilitates healing DCU and may exert anti-inflammatory effects through the up-regulation of miR-146a-5p. Furthermore, it revealed that BMSCs combined with PNS up-regulated the expression of miR-146a-5p most significantly. This implies that both PNS and BMSCs could inhibit inflammatory responses, whereas the combination of both has a stronger anti-inflammatory effect.

The miR-146a-IκBα/NF-κB-IRAK1/TRAF6 axis is a vital signaling pathway that regulates the inflammatory response to trauma repair and is stimulated in the inflammatory microenvironment [47]. Bi et al. found that the inflammatory genes IRAK1 and TRAF6 were positively regulated by NF-κB-related pathway in diabetic wounds, which was consistent with our findings [48]. In addition, upon exposure to inflammatory factors or other stimuli, inhibitors of the NF-κB kinase complex were activated and phosphorylate IκBα, which was degraded by the proteasome to release NF-κB, which then bound to homologous DNA response elements in the target gene promoter to induce expression [49]. NF-κB p65 in DCU was found to be positively correlated with IκBα protein expression, which revealed that inflammatory factors in DCU phosphorylated IκBα and stimulated NF-κB release in the trabeculae, thus increasing the inflammatory response and releasing more inflammatory factors. In addition, our results showed that PNS combined with BMSCs up-regulated the expression of miR-146a-5p, thus reducing downstream IκBα/NF-κB transcription into the nucleus, targeting the expression of IRAK1 and TRAF6 genes and reducing the inflammatory response, especially with PNS combined with BMSCs to promote wound healing. Thus, all the above results suggest that PNS combined with BMSCs has more significant inhibitory effects on miR-146a-5p-IRAK1/TRAF6-NF-κB pathway.

Although the anti-inflammatory and pro-healing effects of PNS combined with BMSCs on DCU and its related regulatory mechanisms were investigated, there were still limitations in this study that should be further investigated. First, the foot area of rats is small, and it is difficult to prepare for foot ulcers. An ulcer model was built with a larger area of the back instead of the foot, which could not well simulate the trauma microenvironment of human DCU. Second, whether suppressing the expression of miR-146a-5p can resist the anti-inflammatory effects of PNS and BSMCs will be tested in the future. In addition, only in vivo experiments were performed in this study, and cellular experiments and clinical validation are lacking to more strongly support the results of this study. Accordingly, further clinical experiments and in vivo experiments are required in the future to support the findings of this study.

## Conclusion

In brief, the results of this study suggested that PNS combined with BMSCs transplantation may reduce the inflammatory response and facilitate the healing of DCU by up-regulating miR-146a-5p expression, down-regulating IRAK1/TRAF6 targeting and inhibiting NF-κB pathway activation.

## Supplementary Information

**Additional file 1: Table S1**. qPCR primer sequences.

## Data Availability

The data supporting this study are open access and can be found in the corresponding databases described in the paper.

## Abbreviations

DCU: Diabetic cutaneous ulcers
DFU: Diabetic foot ulcer
PNSs: *Panax notoginseng* saponins
BMSCs: Bone mesenchymal stem cells
miRNAs: MicroRNAs
DisGeNET: Disease-gene association database
GO: Gene Ontology
H&E: Hematoxylin and eosin staining
KEGG: Kyoto Encyclopedia of Genes and Genomes
MF: Molecular function
CC: Cellular component
BP: Biological Process
OMIM: Online Mendelian Inheritance in Man
qRT-PCR: Quantitative real-time PCR
TCM: Traditional Chinese Medicine
GEO: Gene Expression Omnibus
KEGG: Kyoto Encyclopedia of Genes and Genomes
IL-1β: Interleukin-1β
TNF-α: Tumor necrosis factor-α
