# Supplementary material for: Study on the regulatory effect of Panax notoginseng saponins combined with bone mesenchymal stem cell transplantation on IRAK1/TRAF6-NF-κB pathway in patients with diabetic cutaneous ulcers
Source: J Orthop Surg Res. 2023 Jan 31;18:80. doi: 10.1186/s13018-022-03467-w (PMC9890888; doi:10.1186/s13018-022-03467-w)
Supplement: Supplementary file 1 — Additional file 1: Table S1. qPCR primer sequences. [file 13018_2022_3467_MOESM1_ESM.docx]

**Additional file 1: Table S1**. qPCR primer sequences

| Name | Primer | Sequence |
| --- | --- | --- |
| Rat GAPDH | Forward | 5‘- CTGGAGAAACCTGCCAAGTATG -3’ |
|  | Reverse | 5‘- GGTGGAAGAATGGGAGTTGCT -3’ |
| Rat IRAK1 | Forward | 5‘- CTGGGTTATGTGCCGTTTCTAC -3’ |
|  | Reverse | 5‘- GCCAGGCTGTAATGATGTCCC -3’ |
| Rat TRAF6 | Forward | 5‘- ATCTGCTTGATGGCTTTACGG -3’ |
|  | Reverse | 5‘- CAGCCTTTATTTGGACACTTTACC -3’ |
| Rat IL-1β | Forward | CCAAACCTCTTCGAGGCACA |
|  | Reverse | AGCCATCATTTCACTGGCGA |
| Rat IL-6 | Forward | CCCAACTTCCAATGCTCTCC |
|  | Reverse | AGGTTTGCCGAGTAGACCTC |
| Rat TNF-α | Reverse | CGTCGTAGCAAACCACCAAG |
|  | Forward | CCCTTGAAGAGAACCTGGGA |
| Rat U6 | Forward | 5‘- CTCGCTTCGGCAGCACA -3’ |
|  | Reverse | 5‘- AACGCTTCACGAATTTGCGT -3’ |
| Rat miR-146a-5p | Forward | 5‘- ACACTCCAGCTGGGTGAGAACTGAATTCCA |
|  | Reverse | 5‘- CTCAACTGGTGTCGTGGAGTCGGCAATTCAGTTGAG AACCCATG |
